# Recent Developments in Antimicrobial Polymers: A Review

**DOI:** 10.3390/ma9070599

**Published:** 2016-07-20

**Authors:** Madson R. E. Santos, Ana C. Fonseca, Patrícia V. Mendonça, Rita Branco, Arménio C. Serra, Paula V. Morais, Jorge F. J. Coelho

**Affiliations:** 1 CEMUC, Department of Chemical Engineering, University of Coimbra, Coimbra 3030-790, Portugal; madhsonricardo@gmail.com (M.R.E.S.); ana.clo.fonseca@gmail.com (A.C.F.); patmend@gmail.com (P.V.M.); armenio.serra@gmail.com (A.C.S.); 2 CEMUC, Department of Life Sciences, University of Coimbra, Coimbra 3001-401, Portugal; rsrbranco@gmail.com (R.B.); pvmorais@ci.uc.pt (P.V.M.)

**Keywords:** antimicrobial polymers, biocide polymers, multidrug resistant microbes

## Abstract

Antimicrobial polymers represent a very promising class of therapeutics with unique characteristics for fighting microbial infections. As the classic antibiotics exhibit an increasingly low capacity to effectively act on microorganisms, new solutions must be developed. The importance of this class of materials emerged from the uncontrolled use of antibiotics, which led to the advent of multidrug-resistant microbes, being nowadays one of the most serious public health problems. This review presents a critical discussion of the latest developments involving the use of different classes of antimicrobial polymers. The synthesis pathways used to afford macromolecules with antimicrobial properties, as well as the relationship between the structure and performance of these materials are discussed.

## 1. Introduction

Nowadays, microbial infections are a great concern because they are one of the main primary causes of death worldwide, especially in healthcare institutions, where people are generally more vulnerable [1,2,3]. This fact is mainly due to the persistence of potentially pathogenic microbes (bacteria, viruses and fungi) in several locations, such as textiles, healthcare products, medical devices, water purification systems, sanitation facilities, among others [4,5]. Particular relevance is given to the medical field, in which healthcare infections represent the majority of the adverse events occurring in hospitals. Moreover, most patients exposed to the microorganisms are already in poor health conditions and, therefore, are unable to resist further disorders and other additional complications. For instance, serious infections derived from the use of long-term medical implants (e.g., catheters) can be caused by bacteria that are resistant to different antibiotics [6]. Indeed, the increasing growth of multidrug-resistant microbes, along with the fact that they can easily spread to different environments and by different ways (air, soil and water), makes the prevention of antimicrobial infections very challenging [7]. 

Usually, it is possible to kill microorganisms by sterilization through heat or ionizing radiation [4] or by chemical disinfection (e.g., hypochlorite) [5]. However, these strategies have a short-term action, since the materials or surfaces can be contaminated again after exposure to the pathogens. In addition, disinfectants can both negatively affect the environment and contribute to the growth of resistant microbial strains. Taking into account these limitations, enormous efforts have been made to develop materials that can act as complements to the disinfectant action. Polymers with antimicrobial activity can be also used for the direct elimination of microorganisms as antibiotics. On these matters, the design and synthesis of antimicrobial polymers have gained increasing attention by the scientific community as a safe and effective strategy to combat multidrug-resistant microbes. 

**Figure 1:**
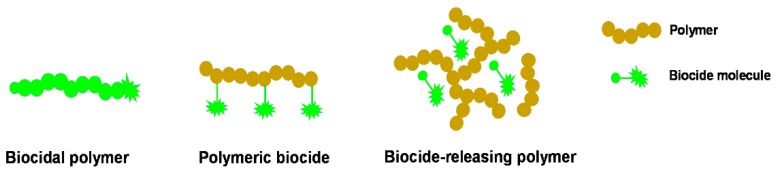
Schematic representation of the different types of antimicrobial polymeric systems (adapted from [8]).

The use of polymers as antimicrobial agents presents several advantages, since these products usually exhibit long-term activity and limited residual toxicity, are chemically stable, non-volatile and do not permeate through the skin [4,7]. According to the type of polymeric system, antimicrobial polymers can be classified into three categories [8] (Figure 1): (i) biocidal polymers, which are polymers with intrinsic antimicrobial activity; (ii) polymeric biocides, which are based on polymer backbones with biocide molecules attached; and (iii) biocide-releasing polymers, which consist of polymers loaded with biocide molecules.

Polymers with intrinsic antimicrobial activity are usually based on polycations, which are able to kill the microbes by action on their negatively-charged cell membrane [9]. Common cationic groups found in these polymers are quaternary ammonium, quaternary phosphonium, guanidinium or tertiary sulfonium [9,10,11]. The use of biocidal polymers is very advantageous considering the design of antimicrobial surfaces, since no harmful biocides will be released to the environment. Concerning polymeric biocides, these are prepared by the attachment of several known biocide molecules to a polymer. Usually, these materials present lower efficacy than the free biocide molecules, due to the steric hindrance created by the polymer backbone [8]. Finally, biocide-releasing polymers are based on polymer matrixes loaded with biocide molecules, which can be entrapped using different methods, or polymers containing biocides attached by cleavable linkages [12,13]. This antimicrobial approach presents an excellent efficacy due to the high concentration of biocide achieved during the release and closeness to the target cells. However, the toxicity associated with the biocide molecules is a serious disadvantage associated with this strategy, and their efficiency reduces significantly with time. 

Regardless of the type of polymeric system adopted, when designing efficient antimicrobial polymers, the polymer should have several important features, such as non-toxicity; stable in long-term applications; stable during the required storage time and at the conditions of its targeted application; and biocidal effect on the broadest microbial spectrum possible. In addition, the polymer synthesis should be simple, straightforward and inexpensive [4]. 

This review covers the most important developments in the start-of-the-art in the field of antimicrobial polymers over the past four years. The information is organized according to different classes of antimicrobial polymeric systems, in which the design and mechanism of action of these polymers is discussed. New approaches based on the use of reactive oxygen species (ROS) are also described as future perspectives in this area. Different aspects of antimicrobial polymers have been covered in past reviews by other authors [14,15,16].

## 2. Most Relevant Microbial Threats to Health and Antimicrobial Evaluation Methods

Microorganisms are everywhere, and their presence affects the environment where they are growing, which can be beneficial (sometimes essential and useful) or harmful. Their universal presence and, particularly, their ability to induce human microbial infection has led to the need for controlling by antimicrobial agents or antibiotics. Antibiotics, which were discovered in the middle of the nineteenth century, successfully reduced the mortality caused by infectious diseases, and they are divided into different classes according to their principal mechanism of action [17].

In recent years, the main concern is the focus on infections caused by multidrug-resistant bacterial pathogens, and this issue has become central to public healthcare systems. Indeed, misuse of antibiotics and several other factors has led to, over the past 30 years, the emergence of a number of resistant bacterial strains, including *Staphylococcus aureus*, *Neisseria gonorrhoeae*, *Escherichia coli* and *Mycobacterium tuberculosis*.

In the United States, antibiotic-resistant bacterial infections add $20 billion to total healthcare costs and $35 billion in costs to society [1]. The Infectious Diseases Society of America has highlighted a fraction of antibiotic-resistant bacteria, the group *Enterococcus faecium*, *Staphylococcus aureus*, *Klebsiella pneumoniae*, *Acinetobacter baumanni*, *Pseudomonas aeruginosa* and *Enterobacter* species—acronymically dubbed “the ESKAPE pathogens”—capable of “escaping” the biocidal action of antibiotics and mutually representing new paradigms in pathogenesis, transmission and resistance [18]. They are responsible for a substantial percentage of nosocomial infections in modern hospitals and represent the vast majority of isolates whose resistance to antimicrobial agents presents serious therapeutic dilemmas for physicians [19,20].

Fungi comprise a large group of microorganisms that can constitute a serious threat for animals and plants. Analysis of the literature and data of disease monitoring has revealed positive trends in pathogenic fungi worldwide and also suggest future increasing of fungal threats under current conditions [21]. Fungal diseases represent an important threat to public health mainly due to: (i) opportunistic infections caused by *Cryptococcus* and *Aspergillus* species; (ii) hospital-associated infections, for instance, caused by a strain of *Candida* yeast; and (iii) community-acquired infections caused by fungi that live in the environment, such as some *Coccidioides*, *Blastomyces* and *Histoplasma* species [21].

The resistance to antifungal agents has been much less studied than antibacterial resistance. However, the current increasing of fungal infections intensified the exploration of innovative, safer and more efficient agents to combat fungal infections.

Multidrug-resistant (MDR) is prevalent in nature [22], and the strategy of eliminating resistance genes makes no sense because the natural function of most resistance genes is not MDR [23]. Therefore, there is an urgent need to create and employ unconventional strategies for the development of antimicrobial products to tackle the rising of global threats imposed by the spread of antimicrobial resistance [24].

### 2.1. Antimicrobial Resistance

Antimicrobial resistance can be gained by different groups of organisms (virus, parasites, bacteria, fungi) [25]. Resistant microorganisms are able to withstand attack by antimicrobial drugs. In bacteria, resistant traits may be exchanged between organisms through conjugation, transduction, transformation or biofilm formation and can spread to the entire colony the resistance for a specific drug [26].

The numerous mechanisms of resistance can be classified as either inherent (natural) resistance or acquired resistance [27]. Inherent (natural) resistance occurs when bacteria are intrinsically resistant to therapeutic agents. Acquired resistance, through the gain of genetic capacity conferring resistance, by chromosomal mutations or better regulation of resistant genes, can lead to bacteria with MDR. Multidrug resistance can include resistance to antibiotics, such as β-lactams, glycopeptides, aminoglycosides, quinolones, sulfonamides, macrolides, linezolid, rifampin and tetracyclines [28].

With respect to fungal infections, the available antimicrobials can be included into three groups based on their site of action: azoles, which inhibit the synthesis of ergosterol; polyenes, which interact with sterols of fungal membrane; and fluoropyrimidine analogs, which inhibit acid nucleic synthesis [29]. The widespread use of all of these classes of antifungal compounds to treat infections or control fungal dispersion leads to the inevitable consequence of antifungal resistance. This resistance may be the result of improved of fungal basic mechanisms, namely reducing the accumulation of the drug within the cell, decreasing the affinity of the drug for its target and changes in the metabolism of the cell.

### 2.2. Bacteria and the ESKAPE Group Concept

The microorganisms primarily reported as being resistant were referred to as ESKAPE pathogens, accentuating their capacity to “escape” from routine antimicrobial regimes [30]. This group includes Gram-negative and Gram-positive microorganisms. In Gram-positive organisms, the main component of the cell wall is peptidoglycan, present in multiple layers. In Gram-negative bacteria, an outer membrane, composed mainly of lipopolysaccharide (LPS), overlaps a thin layer of peptidoglycan.

Among the most challenging MDR organisms of this group are the methicillin-resistant *S. aureus* (MRSA) and vancomycin-resistant MRSA strains. *S. aureus* is a Gram-positive coccal bacterium appearing in clusters, producing the enzyme catalase that can be found in the body of 20% of the human population. *S. aureus* MRSA is actually resistant to an entire class of penicillin-like antibiotics called beta-lactams, and in 2002, one strain was detected to be resistant to vancomycin [31]. Another concern is the microorganism *Enterococcus faecium*, a Gram-positive spherical cell occurring in pairs or chains. Consistently identified as the third most frequent cause of nosocomial bloodstream infection (BSI) in the United States, enterococcal BSIs remain a significant health problem [32]. *Enterobacteriaceae* are a large family of Gram-negative microorganisms. They are rod-shaped, facultative anaerobes and most lacking cytochrome C oxidase. Extended-spectrum beta-lactamase (ESBL)-producing *E. coli* and *Klebsiella* species continue to increase in frequency and severity. *A. baumannii* is a pleomorphic aerobic Gram-negative bacillus. It preferentially colonizes in moist environments. The incidence of infections due to MDR *Acinetobacter* species continues to increase globally [33]. *P. aeruginosa* is a Gram-negative, rod-shaped, bacterium, obligate respirer that has an incredible nutritional versatility. It is a very ubiquitous microorganism that has been found in many different environments. Rates of infection due to resistant *P. aeruginosa* are increasing, as does resistance to both the quinolones (fluoroquinolone) and carbapenems antibiotics [34].

### 2.3. Fungi

Yeasts and molds are ubiquitous in distribution, and many fungi have been isolated from different environments, including indoor environments, due to their ability to grow on almost all natural and synthetic materials [35].

Important factors such as moisture, temperature, pH and nutrients affect the growth of fungi. Some, such as *Aspergillus niger* and *Penicillium funiculosum*, can grow at pH = 2 and below [36]. In terms of nutritional requirements, fungi are the most versatile organisms. They are able to absorb and metabolize a wide variety of carbon sources, such as monosaccharides, disaccharides, polysaccharides, organic acids, lipids and insoluble carbohydrates or very complex hydrocarbons, such as lignin [37]. 

Among the large number of identified species of fungi that are in contact with humans, less than 50 are described in epidemiologic studies. Moreover, reports related to emerging infections have identified important emerging fungi pathogens, such as *Candida albicans*, *Cryptococcus neoformans*, *Pneumocystis jirovecii* [38] and, more recently, *Coccidioides* and *Aspergillus* species [39].

Toxic products of mold growth, for instance microbial volatile organic compounds or microbial volatile break down products, as well as spores and hyphal fragments are recognized as effective sources of disease symptoms [40]. In specific conditions, some fungi strains are also producers of harmful secondary non-volatile metabolites called mycotoxins, such as aflatoxins, by *Aspergillus flavus* and *Aspergillus parasiticus* [41].

### 2.4. Mechanisms of Microbial Cell Death Induced by Antimicrobials

#### 2.4.1. Reactive Oxygen Species-Induced Cytotoxicity

The general knowledge of the potential mechanisms of ROS damage comes from studies in *E. coli*. Superoxide radicals (O_2_·^−^) and hydrogen peroxide (H_2_O_2_) are produced in the cytoplasm when oxygen randomly collides with exposed flavins of cellular redox enzymes [42].

The 4Fe–4S clusters of many proteins are the main targets of ROS damage, but other enzymes are often affected. These protein damages frequently result in metabolic defects that could be dramatic to the cell survival. When iron-sulfur clusters are injured, the iron ion is released and is able to react with H_2_O_2_, in a Fenton reaction, resulting in the production of hydroxyl radical (OH·). All ROS enforce harsh damage to several biomolecules, mostly nucleic acids, proteins and lipids [42,43]. In the case of DNA, the ROS species attacks the sugar group, pyrimidine and purine bases yielding single- and double-strand breaks, several adducts and cross-links to other molecules [44]. The main damages in protein involve oxidation of sulfhydryl groups, modification of prosthetic groups or metal clusters, protein-protein cross-linking and protein carbonylation [45,46], causing inactivation of crucial enzymes, compromising the cell viability. Lipids are also important targets of ROS. The reactive species can easily attack polyunsaturated fatty acids in membranes, leading to lipid peroxidation [47]. This event has a deleterious effect to the cells due to a decrease in membrane fluidity.

Recently, emphasis has been put on the study of stimuli-responsive polymers. Different materials, such as metal and metal oxide nanoparticles and polymeric nanomaterials, that can undergo chemical structure change, biochemical, or physical property variations in response to environmental stimuli are capable of generating ROS and consequently induce cytotoxicity [48,49].

#### 2.4.2. Microbial Cell Envelope Disruption

The bacterial cell envelope is composed of the plasma membrane and cell wall. The latter is essential for prokaryotic cell integrity, reproduction, virulence expression or other functions and can have different structures, with peptidoglycan covered or not with an outer membrane. One of the strategies used to kill bacteria is to disrupt their membranes through the interaction of electrostatic forces between positive residues and negative charges exposed on cell surfaces. Antimicrobial peptides (AMPs), as well, have been suggested to be driven to the cell surface by their composition, acting at this cell level. One mechanism proposed to explain AMPs’ activity suggests that peptides form transmembrane pores by their direct insertion into the lipid core of the target membrane. Others suggest that AMPs intersperse the lipid membrane and others that they cover the membrane, leading to its disruption [50].

Cationic hydrophobic polymers used as antimicrobial coating materials have inhibited both bacteria and human pathogenic fungi species. The cationic polymers interact with the bacterial and fungal cell membrane, disrupting the integrity of the lipid membrane, affecting the transport of compounds across the membranes and, finally, leading to cell death [51].

Since peptidoglycan is not found in eukaryotic cells and has a main role in cell integrity, compounds that inhibit its synthesis are interesting targets for therapeutics. Peptidoglycan destabilization was achieved by poly(acrylic acid)/poly(vinyl alcohol) fibers, the mechanism of which was the withdrawal of the calcium bound to the cell wall [52]. One of the strategies followed by several drugs is interfering with the synthesis of the peptidoglycan. An important precursor of the peptidoglycan synthesis is the lipid II, which was shown to be an attractive target of antibacterial compounds, such as AMPs [53]. In addition, a recent study also demonstrated that the precursors of peptidoglycan, lipids III and IV, are also targets. As an example, nisin and other AMPs bind the lipids interfering with teichoic and lipoteichoic acids biosynthesis [50]. Some products when interfering with the peptidoglycan form at the same time as transmembrane pores [54].

The fungal cell wall contains some unique compounds, such as mannan, chitin and α- and β-glucans. As these components are not shared with other organisms, they have been identified as potential main targets for therapeutic purposes. Like antibacterial AMPs, antifungal peptides can kill fungi by targeting their cell wall, for example through peptide binding to chitin. This peptide-wall binding ability allows the easy access of the peptide to target fungal cells. The fungal peptides show their lethal effect by disrupting the integrity membranes, by increasing membrane permeabilization or by forming pores [55]. Chalcones and their derivatives have also revealed interesting antifungal properties against the group of dermatophytes. These compounds exhibits their action through inhibition of two important enzymes, β(1,3)-glucan and chitin synthase, which catalyze the synthesis of the major polymers of the fungal cell wall, β(1,3)-glucan and chitin, respectively [56].

#### 2.4.3. Inhibition of Nucleic Acid and Protein Synthesis

If the antimicrobial drugs transverse bacterial outer and inner membranes, it is possible that they can accumulate in the cytoplasm and target the nucleic acids. Inside the cell, the substance can bind to DNA and RNA inhibiting their synthesis by inhibiting the DNA-topoisomerase complexes [57]. Indolicidin appears to act by promoting significant membrane depolarization and inhibiting DNA synthesis [58,59]. Polyhexamethylene biguanide (PHMB) enters cells and selectively condenses bacterial chromosomes [60]. Heavy metals inactivate cellular proteins by combining with some component of the protein. Mercury is an example, by inactivating enzymes that contain sulfhydryl (–SH) groups.

In fungal cells, cationic amphipathic peptides, such as magainins, revealed a second antifungal action mode, in addition to its membrane-lysis mechanism, which consists of interfering with the DNA integrity of fungi cells [61].

### 2.5. Evaluation of Antimicrobial Activity

#### 2.5.1. Indicator Organisms for Antimicrobial Characterization

A common group of microorganisms is usually considered for analyzing the antimicrobial polymer activity, as representatives of organisms with similar characteristics. Clinical & Laboratory Standards Institute (CSLI) suggest groups of microorganisms that should be considered for routine testing of antimicrobial agents. The microorganisms include both the Gram-negative and the Gram-positive bacteria, as well as some yeasts and molds from the fungi kingdom. Usually, the Gram-negative type are *E. coli* (USP 51 [62], optional in several tests), *P. aeruginosa (*ASTM E2180-07 [63], USP51, AOAC 964.02 [64], optional in several tests), *Klebsiella pneumoniae* (ASTM E2180, ASTM E 2149-13a [65], AATCC TM174 [66], AATCC TM147 [67], AATCC TM100 [68]) and *Salmonella choleraesuis (*AOAC 955.14 [69], optional in several tests); the Gram-positive bacteria are *S. aureus (*ASTM E2180, AATCC 100 [68], USP 51, AATCC 174, AATCC 147; AOAC 955.15 [70]) and several species of *Streptococcus* (Iranian Research Organization for Science and Technology-IROST). Representative of the filamentous fungi are *A. niger (*USP 51, AATCC 174, AATCC TM30 [71]) and *Aspergillus flavus*, and the yeast forms of fungi, such as *C. albicans (*USP 51, ASTM G21-15 [72], ASTM D4300-01 [73]). Several standard procedures and guidelines for evaluating the antimicrobial activity of materials are available [74]. 

#### 2.5.2. In Vitro Antimicrobial Activity Assay

Methodologies for evaluating the antimicrobial activity are needed each time that it is necessary to evaluate the materials’ safety or to verify the efficiency of the antimicrobial modification processes. Testing antimicrobial properties means testing the microorganisms’ ability to survive under the effect of a given antimicrobial, at a determined concentration and for a certain time period. With regard to terminology, resistance to bacteria is deemed antibacterial and to fungi is antimycotic [75]. A researcher must exercise judgment in selecting a test method for a specific antimicrobial in order to ensure meaningful and reproducible results. Each procedure takes into consideration the specific antimicrobial under analysis and an indicator microorganism representing, according to its characteristics, a group of organisms (see above).

There are a number of classical microbiological procedures that were adapted by CLSI in a standardized form, to reach specific objectives on the evaluation of the antimicrobial activity of the materials [76]. When the objective is assessing the number of microorganisms and their shifting, procedures based on microscopy can be used. By microscopy (optical, electron, fluorescence), bacteria and fungi are counted as particles. The microbial cell morphology can be also observed and can be related to the physiological state of the cell. The plate counting method determines the number of bacterial or fungal cells able to grow in a specific growth medium, and this is reported as colony forming units (CFU) [77]. Additionally, for fungal cells, other conventional methods include the quantification of ergosterol, which is a component of the fungal cell membrane. Moreover, metabolites such as mycotoxins are also often used for the quantification of fungal population and growth. 

The agar diffusion test or the Kirby–Bauer disk diffusion method [78] can measure antimicrobial potency with solid media that clearly show areas of growth inhibition. Assessing antimicrobial activity on agar plates provides semi-quantitative information on the efficiency of the antimicrobial against a given microorganism, but also on the diffusibility of the antimicrobial agent from the material in testing. After a chosen period of incubation, the area of bacteria growth is observed, and the zone of inhibition around the material tested is measured. Modifications of this technique, as well as diffusion assays are also used. 

Evaluation of the microorganism growth by optical density (OD) is followed by UV-VIS spectroscopy. The amount of organisms present in a cultivation medium impacts the turbidity of the medium. Unfortunately, this considers both living and dead cells that did not lose the integrity of their membranes, which can significantly influence the result. 

In the broth dilution procedure, successive dilutions of the antimicrobial under test are performed and dispensed in equal amounts, in tubes of a standardized suspension of indicator organism [79]. Tubes are evaluated, after incubation, for bacterial growth (OD). The minimum inhibitory concentration assay (MIC) is the lowest concentration of a particular antimicrobial needed to inhibit bacterial growth. At this dilution, the test agent is bacteriostatic. This assay is typically performed on planktonic (free floating) bacterial cells. Nowadays, this method is performed in microplates [80].

The minimum bactericidal concentration (MBC) of the antimicrobial can be determined in addition to the determination of the MIC. The minimum concentration of antimicrobial need to kill most (>99.9%) of the viable organisms after incubation during a fixed period is considered the MBC. This method can be also applied to fungi, defining the minimal fungicidal concentration (MFC).

The activity of antimicrobial agents included in materials can also be tested quantitatively by spreading inoculated seeded-agar and, after a specific contact time, enumerating the microorganisms that survived by plating on optimal medium (ASTM E2180 test method). This test enables the determination of the presence of antimicrobial activity and long-term stability of an antimicrobial treatment. For immobilized non-leaching antimicrobial agents, an alternative standard method was developed in shaking flasks (ASTM E2149 test method). 

## 3. Biocidal Polymers

### 3.1. Natural Polymers and Derivatives

Natural polymers represent an important class of materials with interesting properties that make them suitable candidates in several applications ranging from the biomedical field [81] to the packaging industry [82]. In general, natural polymers are biocompatible, biodegradable, available in a large scale and relatively inexpensive [83]. Both for biomedical and packaging applications, the antimicrobial activity is a desirable feature. Until now, chitosan and poly(ε-lysine) are the only known natural polymers exhibiting inherent antimicrobial properties [84].

**Figure 2:**
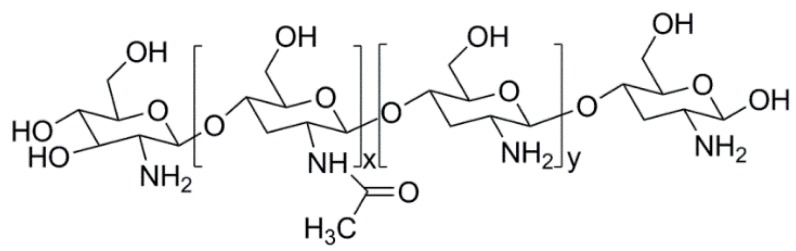
Structure of chitosan.

Chitosan is a cationic polysaccharide obtained from the alkaline deacetylation of chitin, a natural polymer widely found in Nature. It is composed by units of β(1 → 4)-2-amido-2-deoxy-d-glucan (d-glucosamine) and β(1 → 4)-acetoamido-2-deoxy-d-glucan (*N*-acetyl glucosamine), linked together by glycosidic bonds (Figure 2) [85,86,87]. 

The ratio between the d-glucosamine and *N*-acetyl glucosamine units in the chitosan’s polymer chain defines the deacetylation degree, which has a key role in the definition of the majority of the properties of this natural polymer, namely solubility [86], ability to interact with polyanions [88] and antimicrobial activity [89]. Chitosan is insoluble in water, but dissolves under mildly acidic solutions (pKa = 6.5). In these conditions, the –NH_2_ groups of the D-glucosamine units became protonated, turning chitosan soluble [90]. 

The interest in using chitosan as an antimicrobial agent arose when Allan and Hardwiger, in 1979 [91], demonstrated that this polysaccharide has fungicidal ability towards a wide range of fungi. Since then, many other studies proved the antimicrobial activity of chitosan and its derivatives against different microorganisms (e.g., bacteria, fungi and yeasts) [92,93,94]. Typical values of MIC of chitosan against different pathogens can be found in the review of Goy et al. [90]. Chitosan’s application as an antimicrobial agent ranges from the agricultural sector [95], packaging industry [85,96], textile industry [97], to biomedical applications [98]. The role of chitosan as an antimicrobial agent depends on diverse factors. Among those, it is possible to highlight: deacetylation degree [86], molecular weight [97], concentration, physical state (e.g., in solution or in solid state, as fibers, particles or films) [99], and type of microorganism [100], as reviewed by several authors [86,97,99,100]. The mechanism by which this polysaccharide exerts its antimicrobial action remains unclear, and different approaches have been proposed. Many studies evidence that the –NH_3_^+^ groups of chitosan are able to interact with the negatively charged components (e.g., lipopolysaccharides and proteins) of pathogens’ cell walls, changing the permeability barrier properties and inducing the leakage of intracellular components [87,93,101,102,103,104]. Another proposed antimicrobial mode of action relies on the ability of chitosan to pass through the cell membrane and subsequently interact with the DNA of the cell, leading to the inhibition of DNA transcription and protein synthesis [87,105]. It is also believed that chitosan’s chelating capacity could have a crucial role in its antimicrobial capacity. This polysaccharide is able to bind to trace metals (e.g., iron, copper or zinc) at the surface of cell walls, inhibiting the production of toxins or the microorganism growth [87,105,106,107].

Another important trend in the development of antimicrobial materials is the use of chitosan and other polymers, for instance, in the form of blends or nanofilms, produced layer-by-layer (LbL). Hernández-Montelongo et al. [108] prepared nanofilms from chitosan and hyaluronic acid by the LbL assembly technique and tested this material against *Xylella fastidiosa*, a Gram-negative bacteria. The results showed that the nanofilms exhibiting more –NH_3_^+^ groups at the surface were those with the highest antibacterial activity. Gomes et al. [109] used the LbL technique to prepare multilayered materials from chitosan and alginate to coat cotton samples. The antibacterial activity of the coated samples was tested against *S. aureus* and *K. pneumoniae*. The results demonstrated that the treated cotton samples with a five-layered film exhibited the most promising bacteriostatic effect. 

**Figure 3:**
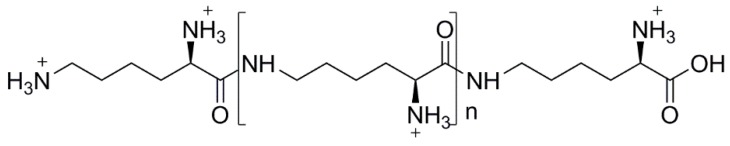
Structure of ε-PL at pH < pI.

Poly(ε-lysine) (ε-PL) is a cationic polyamide consisting of l-lysine units (*n* = 25–30), linked together by the ε-amino and α-carboxyl groups (Figure 3) [84]. ε-PL was identified for the first time by Shima and Sakai, in the late 1970s [110], in the culture filtrates of *Streptomyces albulus*. Nowadays, it is produced industrially by fermentation with mutated strains of *S. albulus* [84]. Other microorganism strains belonging to the genera *Streptomyces*, *Kitasatospora* and *Epichloae* sp. have also shown the capacity to produce ε-PL [84]. This polyamide is biodegradable, non-toxic, water soluble [111] and its antimicrobial properties were already proven against different pathogens, namely Gram-negative and Gram-positive bacteria, yeasts and molds [112,113]. Nevertheless, the mechanism underlying its antimicrobial activity was only studied against *E. coli* strains. When ε-PL is exposed to pH values below its isoelectric point (ca. 9), the –NH_2_ groups became protonated, and two independent studies demonstrated that the –NH_3_^+^ groups are responsible for the antibacterial activity of this polyamide. In the 1980s, Shima et al. [114] studied the mechanism of antimicrobial action of ε-PL towards *E. coli* K-12 cells. The results showed that, firstly, the ε-PL is electrostatically adsorbed at the surface of the bacterium cell wall, followed by the stripping of the outer membrane, which induces an irregular cytoplasm distribution, leading to the cell damage. In the same vein, Ye et al. [115,116] investigated the mechanism of the antibacterial action of ε-PL against *E. coli* O157:H7. The results indicate that the electrostatic interaction disturbs the cell membrane, leading to the formation of pores or structural defects. The disturbance of the cell wall permits the entrance of ε-PL in the cell’s cytoplasm, and as a result, there is the generation of ROS and the interaction with genome DNA, ultimately leading to the cell death.

ε-PL is widely used as an antimicrobial preservative in both food and beverages, but it is known to interact with the anionic components of the products, making them turbid or having a sediment layer. A strategy to overcome this problem is the preparation of complexes of ε-PL with anionic materials. Chang et al. [115] prepared electrostatic complexes from ε-PL and gum arabic, and tested them against acid-resistant spoilage yeasts, viz. *Zygosaccharomyces bailii* and *Saccharomyces cerevisiae*. The results showed that the antimicrobial activity was dependent on the ε-PL and gum arabic ratio, being higher for the complex with the higher amount of cationic polymer. When tested in model apple juice beverages, the complexes with a ratio of gum arabic:ε-PL > 20 have shown good physical properties, without compromising the product appearance. In the same vein, Moschonas et al. [117] studied the effect of caprylic acid, carvacrol and ε-PL, and their blends, in controlling *Salmonella* contamination in frozen breaded chicken products. Overall, the results indicated that both the pristine polymers and their blends could be successfully used as antimicrobial agents for this specific application. 

In a completely different approach, hydrogels made from ε-PL grafted with methacrylamide groups were prepared by Zhou et al. [118] and have shown antimicrobial activity against both bacteria (*E. coli*, *P. aeruginosa*, *Serratia marcescens* and *S. aureus*) and fungi (*C. albicans* and *Fusarium solani*). Interestingly, the hydrogels with higher amounts of grafted methacrylamide showed a potent antimicrobial activity (killing capacity of 99.9%) towards clinically-relevant strains of MSRA and of the antibiotic-resistant *P. aeruginosa*. In general, the hydrogels were more effective at killing bacteria than fungi, but nonetheless, a killing rate near 90% was obtained for the latter. It was also found that this kind of hydrogel can be used to coat contact lenses or other polymeric surfaces with biomedical relevance, giving them antimicrobial properties.

Despite the advantages (e.g., biocompatibility, biodegradability, availability) associated with natural polymers, the control over their structure and properties is sometimes difficult, particularly the reproducibility of the results. On this matter, synthetic polymers are a more convenient alternative to achieve materials with tailor-made properties.

### 3.2. Synthetic Polymers

The most common synthetic biocidal polymers are based on cationic polymers containing either quaternary phosphonium salts (QPS) or quaternary ammonium salts (QAS). This fact is typically accomplished by the synthesis of monomers, which contain biocide moieties (cationic groups) followed by their polymerization or copolymerization with other monomers [5].These materials have been widely used as antimicrobial agents due to some advantages that include higher long-term use, a broad-spectrum of antimicrobial activity and chemical stability when compared to the pristine monomers, as well as lower human cytotoxicity; even LMW QAS compounds tend to be more toxic than primary amine analogues [7,119].

The synthesis of antimicrobial polymers with QAS dates back to the 1970s, when Panarin et al. evaluated the activity of poly((2-methacryloxyethyl)-triethylammonium) [11] against Gram-positive (*S. aureus*) and Gram-negative (*E. coli*) bacteria. The results showed that the antimicrobial activity was enhanced by the increase of the polymeric chain length. As previously observed by other authors for QAS-containing polymers [11], the same observation was done in the 1980s by Ikeda and co-workers et al. [120]. In addition, the authors showed that the antimicrobial activity of the polymers was higher than that of the corresponding starting monomers against Gram-negative bacteria. 

Later, in the 1990s, Kanazawa et al. [121] studied the antibacterial activity against *S. aureus* and *E. coli* of QPS-based low molecular weight (LMW) compounds and their corresponding polymers with different substituents groups (ethyl, n-butyl, phenyl and n-octyl). In addition, the antimicrobial activity of QPS and QAS polymers was compared. For the antibacterial evaluation, it was found that the polymeric phosphonium salt exhibited higher activity than polymeric quaternary ammonium salt. All polymers showed more antibacterial activity than LMW compounds, and it was observed that the compounds with an octyl chain were more active, mainly against *S. aureus*. In this work, the authors could show the contribution of the increase of hydrophobicity to achieve increased antibacterial activity. The same research group has also compared the antimicrobial activity of poly(p-vinylbenzyl tetramethylenesulfonium tetrafluoroborate) samples with different molecular weight values to that of the corresponding monomer [122]. The results showed that the monomer was inactive against *S. aureus* and *E. coli*, even at the highest monomer concentration examined (340 µM). Nevertheless, after the polymerization, the polymeric structures presented antimicrobial activity, which increased with the increase of the molecular weight of the polymers. 

**Scheme 1:**
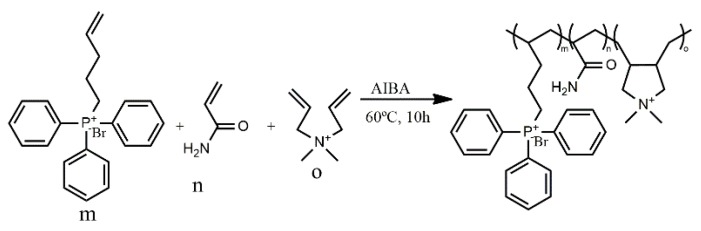
Synthesis route of the cationic random copolymer: (PTPB) (4-penten-1-yl) triphenylphosphonium bromide, (AM) acrylamide and (DADMAC) diallyl dimethyl ammonium chloride using 2,20-azobis(2-methylpropionamidine)dihydrochloride (AIBA) as initiator [127].

It is reported in the literature that polymers with QPS present higher antimicrobial efficacy than similar polymers containing QAS [123,124]. This fact can be explained by the different atomic radii and electronegativity that generate different charge distributions for the corresponding cation, leading to the polarization effect of phosphorus in the phosphonium cations. Ammonium and phosphonium cations present different partial charges due the difference of electronegativity between nitrogen and carbon or phosphorus and carbon. In ammonium cations, nitrogen exhibited a slightly negative charge (−0.5 eV), while the carbons displayed a slightly positive charge (0.3 eV), since nitrogen is more electronegative than carbon. On the other hand, for phosphonium cations, the phosphorus atoms exhibited a positive charge (+1.1 eV), and the adjacent carbons displayed a negative charge (−0.2 eV), as reported by Wang et al. [125] in a study with tetrabutylphosphonium and tetrabutylammonium cations. Due to the negative charge presented by bacterial membranes, the strong polarization of phosphorus allows a more efficient interaction of QPS with the bacteria [126]. It is also possible to prepare biocidal polymers containing both kinds of cationic groups. For instance, Xue and Xiao [127] synthesized a cationic random copolymer, via free radical polymerization (FRP), based on acrylamide (AM), diallyl dimethyl ammonium chloride (DADMAC) and (4-penten-1-yl) triphenylphosphonium bromide (PTPB). The antibacterial activity of the synthesized poly(PTPB-*r*-AM-*r*-DADMAC) (Scheme 1) underwent MIC testing against *E. coli*. The antibacterial activity of the copolymers was also evaluated for samples with a PTPB content ranging from 29% to 55%, and the results showed a higher efficacy for higher contents of the cationic monomer. It is worth mentioning that only copolymers with at least 49 wt % of PTPB showed reasonable antimicrobial activity, as judged by the MIC values (MIC should be lower than 100 ppm) [127]. The same authors tested the antiviral activity against non-enveloped adenovirus (ADV) of a copolymer prepared with 48.7 wt % of the phosphonium cationic segment (PTPB). The results demonstrated a maximum antiviral efficiency of 85.6% using 100 ppm of the copolymer.

In another contribution from the same research group, the authors followed a similar approach and prepared an anti-pathogenic copolymer based on AM, (tributyl(4-vinylbenzyl)phosphonium (QPM)) and [2-(acryloyloxy)ethyltrimethylammonium chloride (ATC)]. The results revealed a decrease of the MIC values in tests against *S. aureus* and *E. coli* with the increase of the phosphonium group (QPM) content in the copolymer, indicating an enhanced bactericidal activity. The virucidal activity against influenza virus and adenovirus revealed that the random copolymer could efficiently inactivate both enveloped and non-enveloped viruses [128]. 

**Figure 4:**
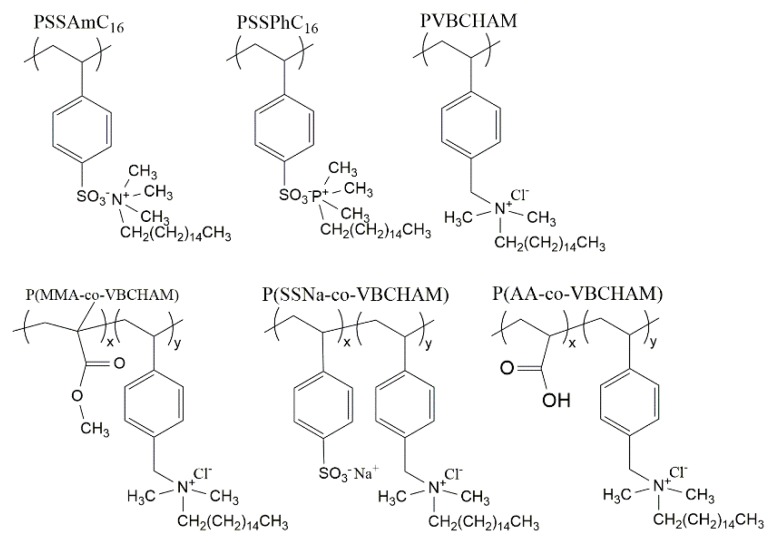
Chemical structure of the homopolymers PSSAmC_16_, PSSPhC_16_, PVBCHAM and the copolymers P(MMA-*co*-VBCHAM), P(SSNa-*co*-VBCHAM) and P(AA-*co*-VBCHAM) [129].

**Scheme 2:**
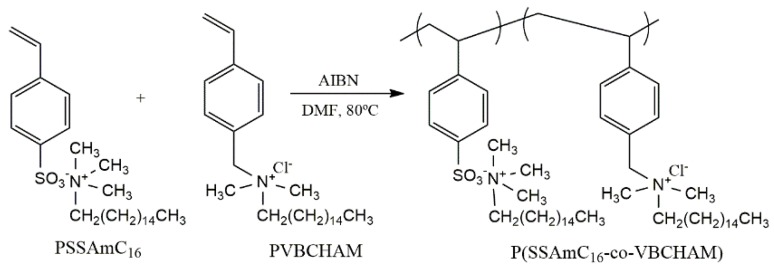
Mechanism for the synthesis of P(SSAmC_16_-*co*-VBCHAM_x_) copolymers [129].

Recently, important research efforts have been done to develop novel polymers with QAS/QPS salts in order to enhance the biocidal efficiency and enlarge the spectrum of antimicrobial and antiviral activity. Kougia et al. [129] investigated a library of homopolymers and copolymers bearing either electrostatically-bound or covalently-attached cationic groups (Figure 4). In addition, several copolymers based on the biocide monomer vinyl benzyl dimethylhexadecylammonium chloride (VBCHAM) were synthesized, using either hydrophilic or hydrophobic co-monomers (Figure 4). The authors found a relationship between polymer chemical structure and the antimicrobial activity (determined as a function of the contact time at 4 °C and 22 °C) against both Gram-negative (*P. aeruginosa* and *E. coli*) and Gram-positive (*S. aureus* and *Enterococcus faecalis*) bacteria. For instance, a higher antimicrobial activity was obtained for the VBCHAM-based copolymers in which acrylic acid (AA) was used as the co-monomer, instead of sodium 4-styrene sulfonate (SSNa) (Figure 3). Moreover, the results have shown that the novel copolymer presenting both covalently-attached and electrostatically-bound quaternary ammonium groups (Scheme 2) provided a remarkable antimicrobial effect [129].

Considering the synthesis of new synthetic biocidal polymers, it could be advantageous to use advanced polymerization techniques, such as the reversible deactivation radical polymerization (RDRP) ones, since they allow the precise control over the molecular weight, structure and composition of the polymers [130,131,132]. On this matter, poly(2-dimethylamino)ethyl methacrylate (PDMAEMA) and poly(dimethylsiloxane) (PDMS) block copolymers, containing a small fraction of 2-hydroxyethyl methacrylate (HEMA), were synthesized by Qin et al. via reversible addition-fragmentation chain transfer (RAFT) polymerization [133]. The block copolymer was obtained by a one-step reaction, using a PDMS-based chain transfer agent to provide control over the polymerization. A post-polymerization modification was made by quaternization of the amino groups of the PDMAEMA segment, with n-octyl iodide, to afford a permanently cationic polymeric segment (QPDMAEMA) in the copolymer. In this study, a series of block copolymers with different chain lengths of QPDMAEMA were prepared in order to evaluate the influence of the charge density and molecular weight of this block on the antimicrobial efficiency of the whole polymer. The results suggested that the antimicrobial activity could be enhanced with the increase of the QPDMAEMA chain length in the copolymer [133].

**Figure 5:**
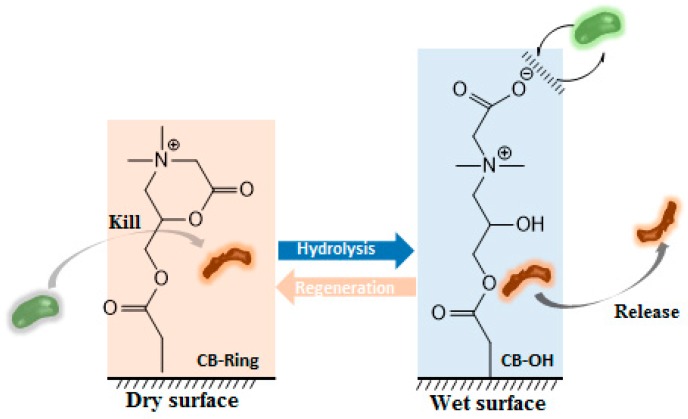
Mechanism of the transition between attack function (CB-ring, killing bacteria) and defense function (CB–OH resists and releases bacteria) (adapted from [135]).

Zwitterionic polymers, which possess the same number of both anionic and cationic groups, have been suggested as a new class of antimicrobial polymers [10,134]. The properties of these polymers enable the adjustment of the polymeric charge density, pH and sensitivity. On this matter, Jiang’s research group reported a “kill-and-release” strategy, which consists of a polymeric surface that is able to kill attached bacteria and then release the dead strains upon the hydrolysis of ester groups of the polymer (Figure 5) [135]. This smart polymer was capable of changing between its two equilibrium states, exhibiting both attack and defending functions. Besides the antimicrobial features, the polymers have also shown non-fouling characteristics [135].

**Figure 6:**
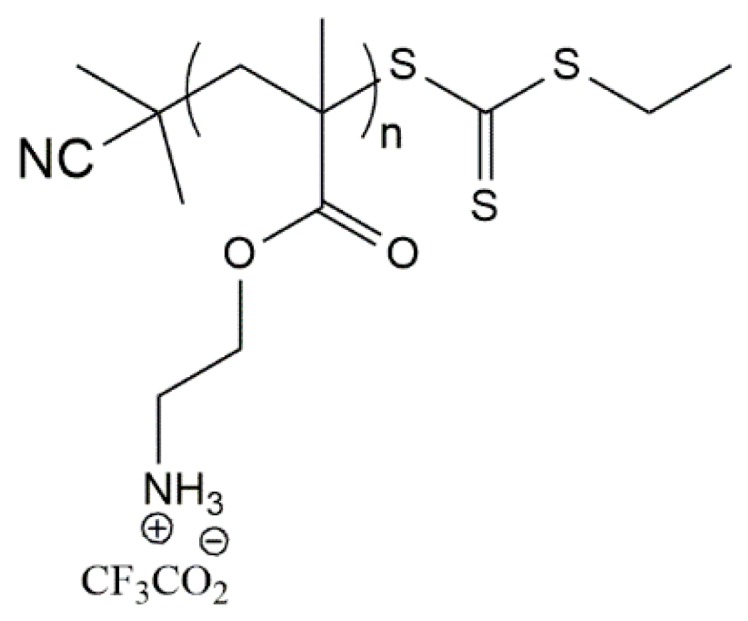
Chemical structure of ammonium ethyl methacrylate homopolymer (AEMPs) [136].

The synthesis of amphiphilic polymers having both hydrophilic cationic and hydrophobic segments is a suitable strategy to afford antimicrobial materials. Recently, the efficacy of primary ammonium ethyl methacrylate homopolymers (AEMPs) (Figure 6) against *S. aureus*, in the nasal environment of cotton rats, was studied [136]. The AEMPs were synthesized via RAFT polymerization of *N*-(tert-butoxycarbonyl)aminoethyl methacrylate, with different contents of primary ammonium groups in the side chains of the polymer. The homopolymers have shown antimicrobial activity against both MRSA and non-MR *S. aureus* and significant reduction in the number of viable *S. aureus* cells in cotton rat nasal infections.

**Scheme 3:**
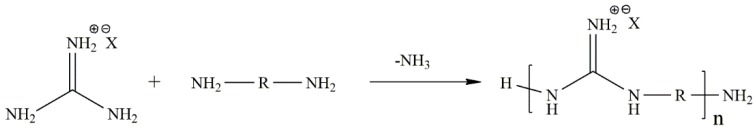
Reaction scheme of the preparation of a synthetic polymer mimic AMPs based on a guanidinium salt [10].

Antimicrobial peptides (AMPs) or host defense peptides are a fraction of the innate immune response and are produced in a variety of eukaryotic organisms [137,138]. Under physiological conditions, the amino acid residues are protonated and then available to interact with the membrane of the bacteria and induce its death [139]. However, the use of AMPs is very limited, since they are extremely expensive, difficult to prepare, can be toxic towards mammalian cells and are chemically instable. On this matter, synthetic polymers that can mimic natural peptides have gained increasing attention as an important alternative to AMPs due to the lower cost of production and milder process conditions [139,140,141]. It is important to note that the efficacy of these polymers can be influenced by several parameters, such as the molecular weight. For instance, polymers with low molecular weight, comparable to that of AMPs, show greater efficacy than monomers or high molecular weight polymers. In addition, a precise amphiphilic balance is required to achieve both high selectivity and antibacterial activity [139,142]. Usually, polymers mimicking natural peptides are based on polyguanidines, polybiguanidines, β-lactams [143], norbonene [144] and also by guanylation reaction with *1H*-pyrazole-1-carboxamidine hydrochloride [145,146], due to their excellent antimicrobial effect, high water solubility, non-toxicity and wide antimicrobial spectrum [10]. Generally, the synthesis is accomplished by reacting chlorocyan, cyanamide, dicyanamide or a guanidinium salt with a diamine (Scheme 3).

**Scheme 4:**
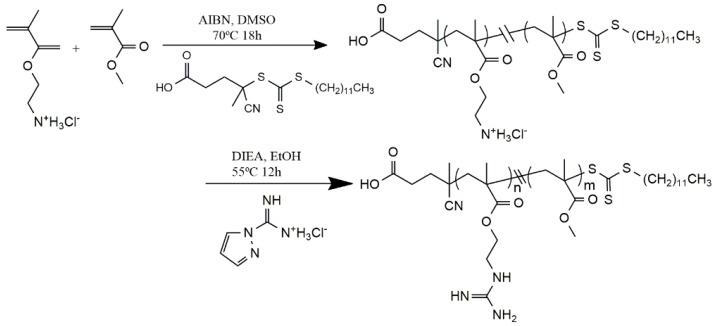
Synthesis of random copolymers containing either amine or guanidine pendant groups [148].

Studies [139,144,146,147] have been reported concerning the antibacterial activity of polymeric structures that mimic AMPs. Locock et al. [148] compared the antimicrobial performance and toxic character of a variety of amphiphilic AMPs with different molar percentages of methyl side chains (MP_methyl_) and a targeted degree of polymerization (DP) synthesized via RAFT (Scheme 4). In this study, the effect against *S. aureus*, *Staphylococcus epidermidis* and *E. coli*, the antifungal effect against *C. albicans*, as well as the hemotoxicity of the copolymers with functionalized amine or guanidine pendant groups were evaluated. The antibacterial and antifungal testing was done determining MIC values using the standard microbroth dilution assay. As a result, the authors could conclude that any polymer could show some effect against *E. coli* attributed to the complexity of the cell membrane in Gram-negative bacteria [149]. In addition, the guanidine polymers were more active against both *S. epidermidis* and *C. albicans*, displaying MIC values in the range of 2–20 µg/mL, while the MIC values for the polymers with the amine groups were considered too high for practical use. Combined results for antibacterial activity, toxicity effect and degree of hemolysis for guanidine copolymers of low to medium molecular weight and 32%–44% of methyl monomer content showed the best results, considered to be an effective antimicrobial with low toxicity effect, in contrast to that results obtained for amine analogues.

**Figure 7:**
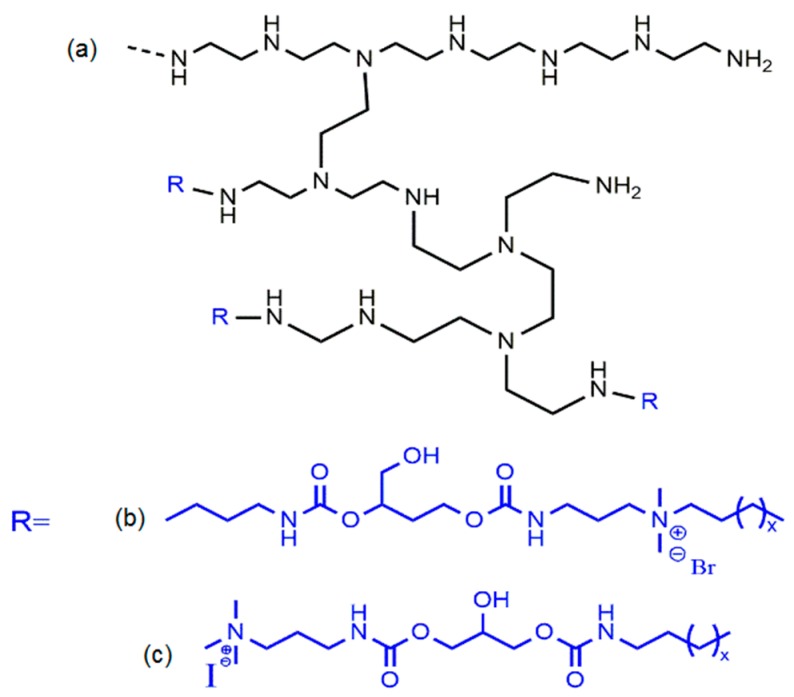
Chemical structures for: (**a**) poly-(ethylene imine); (**b**) alkyl chains directly linked to cationic groups and (**c**) alkyl chains and cationic groups linked by a spacer [150].

**Figure 8:**
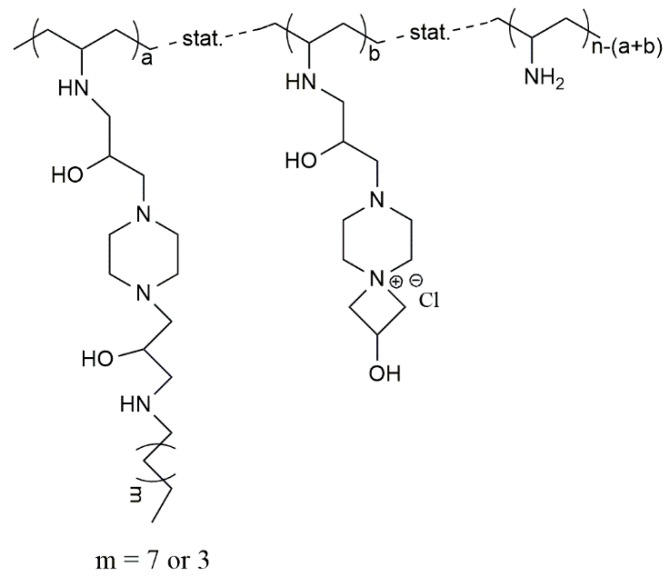
Chemical structure of poly(vinyl amine) with cationic, azetidinium and alkyl groups on the side chain [151].

Möller et al. synthesized antimicrobial poly(ethylene imine)s (Figure 7) using cationic, hydrophobic or amphiphilic monomers to obtain different microstructures [150]. The microstructures containing the hydrophobic segment attached directly to the segment with cationic groups showed higher efficiency against bacteria than the one with cationic groups that are not attached to the alkyl chain [150]. Later, the same group tested multifunctional poly(vinyl amines) (PVAms), with cationic, reactive azetidinium and alkyl chain groups introduced in the side chains of PVAms, against bacteria (Figure 8) [151]. The antimicrobial efficacy of the polymers was improved by increasing the hydrophobic moiety, namely by replacing the hexyl group by the decyl group. In addition, a higher content of cationic groups has also led to a significant increase of the antimicrobial efficiency. The results suggested that the PVAms samples containing 11% of hydrophobic and 5%–14% of cationic groups presented the best antimicrobial efficacy [151].

## 4. Polymeric Biocides

**Figure 9:**
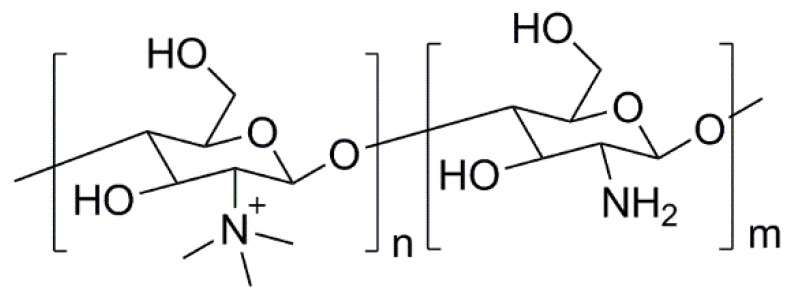
Structure of TMC.

Polymeric biocides consist of polymer backbones in which biocide molecules (e.g., antibiotics) are attached to provide antimicrobial activity. In some cases, by modifying the structure of non-antimicrobial polymers, it is also possible to induce antimicrobial activity. This strategy is commonly used with chitosan. As mentioned before, the antimicrobial activity of chitosan is ruled by the presence of the –NH_3_^+^ groups in its structure. However, this charge “development” only occurs in acidic conditions, impairing the use of chitosan as an antimicrobial agent at neutral pH. To overcome such a drawback, typically, nitrogen atoms in the chitosan’s structure are quaternized, yielding a derivative that is not only permanently charged, but also water-soluble [99,152]. *N*,*N*,*N*-trimethyl chitosan (TMC) (Figure 9) was the first quaternized-chitosan derivative to be developed and demonstrated to have good antimicrobial activity [153]. Since then, many other works have been reported, and it was demonstrated that these derivatives have a greater antimicrobial activity than neat chitosan, as recently reviewed by Martins et al. [99]. This type of derivatives are seen as the most promising due to their permanent charge, but other chitosan derivatives (viz. thiolated derivatives or carboxymethyl chitosans) have also been shown to be promising antimicrobial agents [84,97].

**Figure 10:**
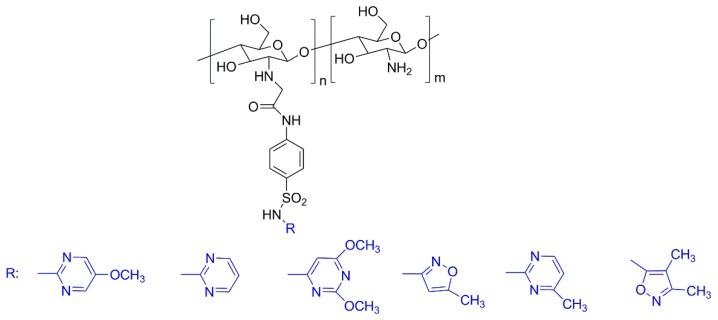
Structures of the sulfonamide-chitosan derivatives prepared by Dragostin et al. (adapted from [154]).

Recently, a considerable number of works regarding the use of chitosan and its derivatives as antimicrobial agents was published. Very recently, Dragostin et al. [154] used chitosan-sulfonamide derivatives (Figure 10) to prepare new wound dressing biomaterials. The antimicrobial activity of the wound dressings was evaluated using both bacteria and fungi strains. Overall, the results revealed that the chitosan-sulfonamide derivatives have greater antimicrobial activity than the neat chitosan, indicating that the introduction of the sulfonamide moiety has a positive effect regarding this property. Moreover, the in vivo studies carried out on a burn wound model induced in Wistar rats showed that the membranes based on such derivatives are more effective in promoting the healing and the epithelization processes than chitosan [154].

**Figure 11:**
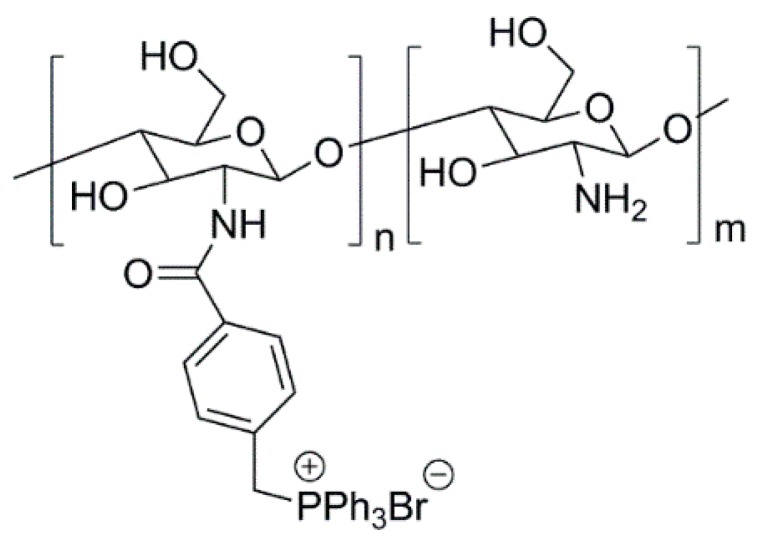
Structure of the chitosan derivatives bearing quaternary phosphonium salts used by Zhu et al. [155].

On the other hand, Zhu et al. [155], prepared a series of water-soluble chitosan derivatives bearing quaternary phosphonium salts (Figure 11). The different derivatives were shown to have antimicrobial activity towards two different bacterial strains (*E. coli* and *S. aureus*). The results have also demonstrated that the antibacterial activity is higher for the derivatives with a higher degree of substitution (DS) (viz., 26.7% and 41.4%). The antibacterial efficacy determined by the LB-agar plate methods for the chitosan derivative with a DS of 41.4% was 99.9% for the *E. coli* strain and 99% for the *S. aureus* strain. For neat chitosan, the antibacterial efficacy did not surpass 40%. Moreover, the chitosan derivative with a DS of 41.4% was shown to have low cytotoxicity towards a human skin fibroblast cellular line, making it an interesting candidate to be used in the biomedical field.

**Figure 12:**
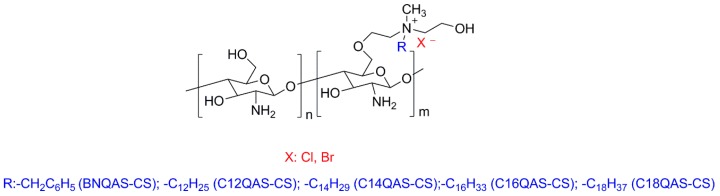
Structures of the *O*-quaternary ammonium salt chitosan derivatives.

In another contribution, Wang et al. [156] developed *O*-quaternary ammonium salt chitosan derivatives (Figure 12) with the ability of being dissolved in aqueous medium and tested them as antimicrobial agents against bacteria and fungi. The results demonstrated that all of the chitosan derivatives were more effective against the microorganisms than the pristine chitosan, for all of the concentrations and times of contact investigated. The best results were obtained for Gram-positive bacteria (*S. aureus*, *α-Hemolytic streptococcus*, *β-Hemolytic streptococcus*) with the C12QAS-CS derivative (Figure 12). The worst results in terms of antibacterial activity were exhibited by the C18QAS-CS derivative (Figure 12). This behavior was attributed to its lower DS and the low antibacterial activity of the C18QAS monomer. Regarding the antifungal effectiveness, it was found that C14QAS-CS and C16QAS-CS were the most promising polymers, with antifungal properties of 100% at concentrations of 100 ppm. However, the in vitro cytotoxicity tests carried out on a mouse alveolar epithelial type II (AT2) cell line showed that the chitosan derivatives have higher cytotoxicity than the pristine chitosan. 

As mentioned before, the solubility of chitosan (or its derivatives) in water is a critical aspect regarding the antimicrobial activity. Nevertheless, Inta et al. [157] managed to prepare a hydrophobic chitosan derivative, viz. dodecenyl succinylated phthaloyl chitosan, with different DS, to be used as an antibacterial agent. The derivatives were used to prepare films and were tested against different bacterial strains, namely *E. coli*, *Bacillus subtilis* and *S. aureus*. The results showed that the chitosan derivative films were more effective at inhibiting bacterial growth when compared to the films based on neat chitosan. 

## 5. Biocide-Releasing Polymers

### 5.1. Nanoparticles Release

Metallic nanoparticles have attracted increased attention as suitable antimicrobial agents. Recently, a variety of different polymers that incorporate these metallic nanofillers have been developed in order to afford materials with biocidal activity for diverse areas, such as healthcare [158], water treatment systems [159], antimicrobial surfaces, dental and medical devices [158]. However, there are also some important issues concerning the safety of the nanoparticles, since they can present health and environmental hazards [160]. The size of the nanoparticles is comparable to that of human cells, and large proteins of the regular human immune system may not work against them to avoid toxicity effects [161].

There are several methods to entrap nanoparticles in the polymeric matrixes. One approach involves in situ polymerization after the addition of the nanofiller to the monomer phase. Another technique consists in the mechanical blending of the polymer matrix in the molten state. This method is typically applied to polymeric materials that are not suitable for in situ polymerization [161]. Anh et al. have reported a new technique for the incorporation of silver nanoparticles (AgNPs) into a polyethylene (PE) matrix using a melt/blending approach to afford PE/AgNPs nanocomposites [162]. The nanocomposites were prepared by mixing the AgNPs powder and PE granules at 170 °C and 60 rpm for 8 min. In this work, the antimicrobial activity was assessed against strains of *E. coli*, *Salmonella typhimurium* and *Bacillus subtilis* [162]. The PE/AgNPs nanocomposites showed significant antibacterial activity during the early growth stage or lag-log phases, even using a concentration of AgNPs as low as 5 × 10^−5^ wt %.

In situ polymerization of polyurethane (PU) in the presence of AgNPs was used to prepare antimicrobial foams for use in low-immunity patients’ rooms and intensive care units [163]. PU/AgNP foams were prepared with four different weight contents of AgNPs (0.0%, 0.1%, 0.5% and 1%) by mixing thoroughly the mass of the silver nanofiller with the polyol blend before the reaction with the isocyanate prepolymer, to afford in situ AgNPs-loaded nanocomposites. The activity was assessed by optical density (OD) assay, and the best result was obtained for the sample with 0.1 wt % of AgNPs, showing the lowest OD values (0.184 for *Klebsiella* sp.; 0.131 for *Staphylococcus* sp.; and ≈0.0 for *E. coli*).

The use of bimetal-dispersed porous polymeric beads is also a possible strategy to enhance the antimicrobial activity of polymer/metal blends [164]. Cu:Ag-based nanoparticles-doped carbon porous beads were prepared by suspension polymerization with phenol and formaldehyde monomers, followed by carbonization and reduction with hydrogen atmosphere. A variety of polymeric beads was prepared with different amounts of Cu and Ag, using the single-metal (Cu or Ag) salts for comparison. The antibacterial effect was determined by the plate counting method using LB-agar medium. The results showed a complete growth inhibition of *E. coli* and *S. aureus* in water during 120 h. The highest antimicrobial activity was observed for the optimized sample incorporating 2 wt % of Cu and 0.2 wt % of Ag. The results indicated that relatively inexpensive Cu is able to partially substitute expensive Ag metal in the polymeric beads. Therefore, this approach is very interesting since it can be used to develop affordable systems for water treatment.

Poly(vinyl pyrrolidone) (PVP) nanofibers loaded with different metallic nanofillers (Ag, Cu and Zn) were prepared in order to assess the influence of the nature of the metal on the antimicrobial activity of the material against *S. aureus* and *E. coli*. [165]. The results showed that the fibers loaded with Ag nanoparticles presented the highest antimicrobial activity and led to a lower amount of viable cells, in comparison with the polymers containing either Cu or Zn [165]. 

Polymer/Cu nanoparticles (NPCu) have been also used due their antimicrobial activity. Composites having NPCu with two very distinct diameters (10 nm (NPCu) and 45 µm (MPCu)) were investigated in terms of the antimicrobial activity and Cu ion release [166]. NPCu and MPCu particles were incorporated in 1% and 5% polypropylene (PP) matrix by a melt mixing process. This study highlighted the importance of the water and the oxygen diffusion through the polymer bulk in the efficiency of the oxidation reactions of the embedded Cu particles. When *Pseudomonas aeruginosa* and *S. aureus* were placed in contact with the samples, the bactericidal effect was found to be proportional to both the Cu ion concentration, particle size and amount used. Samples with 1% PP/NPCu and 5% PP/NPCu showed activity against both bacteria after 30 min, while samples of 1% PP/MPCu and 5% PP/MPCu presented some significant effect only after 90 min of contact with bacteria.

Recently, composites based on chitosan with ZnO nanoparticles immobilized by in situ sol-gel conversion were reported [167]. In this contribution, three solutions with different NaOH concentrations (15%, 30% and 45%) were used to prepare the samples designated as CN15, CN30 and CN45, respectively. The antibacterial activity of the materials was evaluated by the agar well diffusion method [168], using chitosan as the reference material. The inhibition zone of microbial growth was used as a measure of the antimicrobial activity. The antimicrobial activity, evaluated against bacteria *E. coli* and *S. aureus*, was higher for the samples containing ZnO nanoparticles, in comparison with the pure chitosan. PVP-capped ZnO nanoparticles and doped with Fe (1% and 10%) and Mn (1% and 10%) were synthesized using a bottom-up wet chemical precipitation technique [169]. The bactericidal and fungicidal effect was evaluated by using a standard disc diffusion method. The MIC, MBC and maximum fungicidal concentration (MFC) were determined to assess the antimicrobial characteristics of the materials. The results obtained for the three parameters evaluated showed that the sample doped with 10% of Fe and Mn ions was more efficient than the one with 1% of metal content, suggesting that the amount of both Fe and Mn played an important role in the antimicrobial activity of the materials. Other related studies have proposed possible mechanisms of interaction between metallic nanoparticles and microorganisms, such as: the inhibition of bacterial growth by the generation of H_2_O_2_ from the surface of ZnO [170]; the oxidative stress and lipid peroxidation from the production of ROS by ZnO NPs [171]; and the deactivation of the cellular enzymes and DNA of microorganisms [172].

### 5.2. Antibiotics Release

As mentioned before, one of the most prominent problems in healthcare is related to the infectious diseases caused by bacteria, viruses or fungi. Data from the World Health Organization (WHO) indicate that the diseases caused by these pathogens are one of the leading causes of global hospitalization and mortality [173]. Moreover, the appearance of multidrug-resistant bacteria is driven not only by the over-prescription of antibiotics and their inadequate use by patients [174,175], and but also from the intensive use of antibiotics in animal husbandry, agriculture and aquaculture [176,177]. Additionally, issues like systemic toxicity and the short half-life of antibiotics should not be neglected [174,175,178]. Taking into account all of these factors, it is possible to conclude that it is urgent to develop new “anti-pathogen” drugs or to develop strategies that could enhance the bioavailability of the drug, while reducing its side effects [174,175,178]. As mentioned in the previous sections of this manuscript, some polymers have inherent antimicrobial properties and can be used per se as the “anti-pathogen” drug. In fact, the works dealing with such polymers suggest that they can be successfully used against a vast number of pathogens, in diverse applications. Another possible strategy involves the development of effective drug delivery systems making use of polymeric materials [173,175,178,179]. For instance, polymeric particles have been shown to promote a slow and sustained release of antibiotics, while improving their pharmacokinetics and biodistribution. Very importantly, this type of carrier was also shown to facilitate the transport of the drug through cellular and tissue barriers. An excellent recent review about this topic can be found in the literature, where the relevant works in the field, along with their main findings are presented [175,180,181]. In a very recent and interesting contribution, Li et al. [13] prepared polymer vesicles from poly(ethylene glycol) (PEG) and hydrophobic blocks containing enzyme-cleavable self-immolative side linkages. The main objective of this work was to develop a delivery system responsive to the action of enzymes, especially those that are produced by resistant pathogens, viz. penicillin G amidase (PGA) and β-lactamase (*Bla*). The polymer vesicles exhibited the ability of entrapping both hydrophilic and hydrophobic drugs, releasing them in a controlled manner. Very interestingly, the release of the antibiotic from *Bla*-degradable vesicles was triggered in the presence of MRSA, with a concomitant inhibition in the microorganisms’ growth. In addition, the polymer vesicles also demonstrated the capacity to enhance the wound healing process in an in vivo murine model.

Dendritic structures have also proven useful in the delivery of antibiotics as described in the recent review by Kalhapure et al. [181]. In recent years, although less explored than the strategies presented above, antibiotic delivery systems based on hydrogels [182,183,184] and nanofibers or nanofibrous mats [160,185,186,187] have been also used. Gustafson et al. [183] prepared charged hydrogels based on oligo(poly(ethylene glycol)fumarate) and sodium methacrylate (OPF/SMA), by photopolymerization, and used it as a delivery device for vancomycin in surgical applications. The hydrogels have been shown to effectively load the antibiotic, the loading mechanism being dependent on the hydrogel’s charge. The release of vancomycin from this system could be prolonged over four days. Importantly, the antimicrobial activity of the antibiotic was similar to that observed in antibiotic solutions. The authors suggested that this system could be very useful for the local delivery of antibiotic for surgical site infection prophylaxis. 

In a different approach, Steffensen et al. [184] prepared a hydrogel based on silicone and PHEMA by a high-pressure polymerization process. Interpenetrated hydrogel networks were formed when the contents of PHEMA were at least 25 wt %. The hydrogels with 25 wt % PHEMA were loaded with ciprofloxacin, and their antimicrobial activity was evaluated against a strain of *S. aureus*. It was demonstrated that the drug released from the device was effective at inhibiting the bacterial growth. The authors anticipated the usefulness of this hydrogel as part of medical devices, in which the antibiotic action will supplement their functions (e.g., urinary catheters).

As mentioned above, nanofibrous materials have also been recently explored in the delivery of antibiotics. Jalvandi et al. [186] prepared composite nanofibrous mats for the controlled release of levofloxacin (LVF) and tested their antimicrobial efficacy against an *E. coli* strain. The composite materials were composed of mesoporous silica nanoparticles (MSN) loaded with LVF embedded in poly(ε-caprolactone) (PCL) nanofibers and prepared by the core-shell electrospinning technique. The drug release tests, performed in phosphate-buffered saline solution (pH = 7.4), at 37 °C showed a decrease in the LVF burst release of ca. 40%, when the composite mats were used, in comparison with LVF-loaded PCL (LVF/PCL) nanofibers. It was also seen that the composite mats promoted a sustained release of the drug over 13 days. Importantly, the composite mats have been shown to have a high antibacterial activity against *E. coli*, being active during 14 days, whereas the LVF/PCL nanofibers lost their antibacterial activity after seven days. 

In another approach, Son et al. [160] prepared pH-responsive nanofibrous mats from Eudragit EPO (EPO) and Eudragit L100 (L100) through the electrospinning technique. Tetracycline (TC) was used as the model antibiotic. The TC release was shown to be dependent both on the ratio of EPO and L100 in the mats and on the pH of the medium. The release was faster for pH = 6 and for the mats with high contents of EPO. The antibacterial activity of the mats was tested against an *E. coli* strain, and the results showed that the TC-loaded mats were effective at inhibiting the microorganism’s growth, the best results being obtained for the samples with high contents of EPO and at pH = 6. Taking into account the results, the authors anticipate that this type of delivery system could be used, for instance, to locally treat gastric infections.

Guadalupe et al. [185], prepared PCL nanofibers by electrospinning, incorporating a model angiogenic factor (PNF1) and gentamicin, to be used as a dressing for wound healing applications. The use of PNF1 had the main aim of making the wound dressing bioactive, which means that, when applied, the dressing should contribute to vascularization of the new tissue. The results have shown that the presence of this molecule in the dressing improved significantly the proliferation of human microvascular endothelial cells (HMVEC). The nanofibers were coated with chitosan as a method to control the initial drug burst release, to hold moisture and potentiate the antibiotic action. The results indicated that the delivery system was able to promote a sustained release of the drug over 15 days (ca. 60%). The antimicrobial activity tests carried out with an *S. aureus* strain demonstrated that the dipping of the nanofibers with chitosan was essential to inhibit the microorganisms’ growth. Although chitosan could potentiate the antimicrobial activity of the nanofibers, the addition of gentamicin had a key role with respect to the inhibition of the bacterial growth.

**Figure 13:**
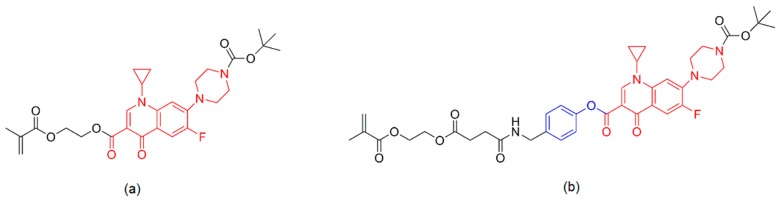
Structures of the prodrug methacrylated monomers: (**a**) prodrug with the aliphatic ester linkage; and (**b**) prodrug with the aromatic ester linkage. In red is represented the structure of ciprofloxacin.

Another strategy to combat bacteria encompasses the covalent linkage of the antibiotic to the polymeric backbone. The interesting advantage of this approach relies on the fact that the release of the active compound can be easily promoted based on the chemical linkages (e.g., ester, urethane, amide) that link the drug to the macromolecule [178]. β-lactams (e.g., penicillin, cephradine), fluoroquinolones (e.g., norfloxacin, ciprofloxacin, levofloxacin), aminoglycosides (e.g., gentamicin) and sulfonamides (e.g., sulfamethoxazole) are examples of different classes of antibiotics that have been covalently linked to polymer backbones [178]. Other approach that has been used encompasses the preparation of a monomer from a specific drug (e.g., norfloxacin [188]) and its polymerization to yield a polymer with antimicrobial activity. In this vein, in a very recent contribution, Das et al. [189] prepared prodrug methacrylate monomers from ciprofloxacin, with two distinct linkages, viz. phenolic or aliphatic ester (Figure 13). The monomers were then copolymerized with poly(ethylene glycol) methacrylate (O950) or chain extended from a poly(O950) macro chain transfer agent (CTA), to yield hydrophilic copolymers or diblock copolymers, respectively. At physiological pH, the diblock copolymers self-assembled into micelles that disassembled upon a pH drop to six. In turn, the hydrophilic copolymers formed unimers whose particle size was independent of the solution pH. The ciprofloxacin release was shown to be dependent on the type of linkage in the prodrug, being faster in the case of phenolic ester linkage (50% after 120 h vs. 50% after 22 days). The antimicrobial activity of the prodrugs was tested against *Burkholderia pseudomallei* in a bacteria-macrophage co-culture model of melioidosis, and MIC values of 6 mM and 0.6 mM were obtained for the aliphatic and phenolic ester prodrugs, respectively. 

The majority of delivery systems for antimicrobials developed until today are essentially used in the delivery of antibiotics, and the works that have been published in the last few years demonstrate the importance of this subject. Nevertheless, some work has also been done in the development of delivery systems for antifungal agents. Detailed information about such systems can be found in recent review literature [190,191,192].

## 6. Polymers Containing Photosensitizers: A New Class of Biocidal Polymers

One novel and promising approach to treat infections involving the presence of different pathogens relies on the use of photodynamic inactivation (PDI) [193] that consists of the local overproduction of ROS to inactivate microbes. This process is mediated by a non-toxic dye or a photosensitizer (PS) and light of low intensity (milliwatt range). When oxygen is present, the PS in its excited state transfers energy or electrons to ground state molecular oxygen, resulting in the production of ROS (singlet oxygen and hydroxyl radicals) that kill microbes. This strategy presents some very interesting features, such as: low risk of resistance development; long-term antimicrobial effect; and elimination of a broad range of microorganisms (Gram-positive and Gram-negative bacteria, fungi, yeast and virus (including HIV)) [194,195]. Plaetzer et al. [196] have bound curcumin to PVP for the PDI of both Gram-positive and Gram-negative bacteria. Using a blue light LED array (E = 33.8 J cm^−2^, λ = 435 nm) resulted in a >6 log10 reduction of viable *S. aureus* after 5 min of incubation. The data revealed that higher incubation periods resulted in lower phototoxicity, probably due to the degradation of the PS. Furthermore, for a PVP-curcumin concentration of 50 µM, the complete eradication of *S. aureus* was achieved. In a very interesting contribution, Parkin et al. [195] studied the incorporation of crystal violet, methylene blue and 2-nm gold nanoparticles into medical-grade silicone. The samples showed antimicrobial activity when exposed to a white light source (intensity of 3750 lux). Interestingly, even in dark conditions, for incubation times of 3 h, contrary to methylene blue and nanogold encapsulated samples, the crystal violet-coated silicones revealed antimicrobial activity. Among the different samples studied, the crystal violet silicone samples encapsulated with 2-nm gold nanoparticles were demonstrated to be the most effective against *Staphylococcus epidermidis*. Liu et al. studied the use polyfluorene derivative containing a quinoline skeleton and quaternary ammonium group-modified side chains (PFPQ) [197]. The authors demonstrated that the binding between the Gram-negative *E. coli* and the polymer was mainly dominated by electrostatic interactions. The killing efficiency was found to be higher than 99% using white light (400–800 nm) with a dose of 18 J·cm^−2^. The antimicrobial properties of PFPQ were attributed to a collaborative effect resulting from the dark toxicity of quinolone and the ROS produced by the polymer backbone under white light.

## 7. Conclusions and Outlook

Microbial infections cause 100,000 deaths annually in the USA alone and affect a broad range of human activities, including food processing, transportation and healthcare services. This very serious problem is expected to get worse during the coming years due to several reasons that include: inappropriate prescription of antibiotics for viral diseases; incomplete patient treatment regimens; and the massive use of antibiotics in livestock feedstuff. If no effective solutions are found in the coming years, most probably, one day, infections will become again the main cause of premature death. 

Antimicrobial polymers could have a decisive role in this global effort to find effective solutions for a problem that affects everybody due to their intrinsic features. Despite the important scientific advances achieved during the last few years, a massive amount of work remains to be done aiming to address major challenges, such as: enhancing the antimicrobial activity; enlarging the range of microbes that can be effectively eliminated by each material; increasing the long-term stability of antimicrobial polymers; eliminating the toxicity associated with some strategies; and developing cost-effective solutions. The line of research involving the use of natural polymers has been mostly focused on chitosan modifications. It will be decisive to understand the mechanisms of action involving the ensuing polymers resulting from the selective modification of this natural polymer. Nevertheless, the limitations associated with the lack of control over the polymer backbone structures that are common to all natural polymers represent a critical issue in this strategy. 

On this matter, synthetic polymers could be a very suitable approach. At the same time, the remarkable progresses achieved in other areas, namely the RDRP methods, will be extremely important to design tailor-made functionalized polymers with enhanced activity. These exciting developments have opened an unprecedented toolbox for the synthesis of polymers with stringent control over the structure, morphology and topology by using radical methods. The judicious design and synthesis of specific macromolecules to afford a clear understanding of the structure/performance relationship is nowadays a reality. For example, it is possible to fine-tune amphiphilic balance in order to enhance both selectivity and antibacterial activity. This research path previously led to the synthesis of a new generation of functionalized antimicrobial polymers with outstanding antimicrobial efficacy. Another promising area involves the use of molecules that can mimic natural peptides as an alternative to AMPs. The appearance of new AMP-like structures will open the door to further important developments involving their use as functionalization residues. 

Another strategy deals with the use of molecules with biocide features that can be released from the polymer matrix. However, constraints associated with the potential resistance development when antibiotic molecules are involved or safety issues associated with the use of releasing nanoparticles could limit the potential of these strategies in the future. Recently, the antimicrobial PDI emerged as a very promising strategy with high efficiency, a very low risk of resistance development and the capacity to eliminate a wide range of microorganisms. One should expect significant developments of this approach fueled by the intrinsic advantages when compared to other strategies. It will be extremely important to enhance the efficiency of these systems by developing new PS with higher performance at an affordable cost. 

Last, but not the least, the discovery of effective antimicrobial polymers will be possible only through the effective sharing of knowledge between the areas involving the synthesis of polymers and microbiology.

## Abbreviations

The following abbreviations are used in this manuscript:

AA	Acrylic acid
ADV	Non-enveloped adenovirus
AEMPs	Primary ammonium ethyl methacrylate homopolymers
AgNPs	Silver nanoparticles
AIBA	2,20-azobis(2-methylpropionamidine)dihydrochloride
AM	Acrylamide
AMPs	Antimicrobial peptides
APMA	Methacrylamide hydrochloride
ATC	2-(acryloyloxy)ethyltrimethylammonium chloride
*bla*	β-lactamase
BSI	Bloodstream infection
CFU	Colony forming units
CTA	Macro chain transfer agent
DADMAC	Diallyl dimethyl ammonium chloride
DS	Degree of substitution
EPO	Eudragit EPO
ESBL	Spectrum β-Lactamase
FRP	Free radical polymerization
GPMA	3-guanadinopropyl methacrylamide
HEMA	2-hydroxyethyl methacrylate
LB	Lysogeny broth
LbL	Layer-by-layer
LMW	Low molecular weight
LPS	Lipopolysaccharide
LVF	Levofloxacin
MBC	Minimum bactericidal concentration
MDR	Multidrug-resistant
MFC	Minimal fungicidal concentration
MIC	Minimum inhibitory concentration assay
MRSA	Methicillin-resistant *Staphylococcus aureus*
MSN	Mesoporous silica nanoparticles
NPCu	Polymer/Cu nanoparticles
OD	Optical density
OPF/SMA	Oligo(poly(ethylene glycol)fumarate) and sodium methacrylate
PCL	Poly(ε-caprolactone)
PDMAEMA	Poly(2-dimethylamino)ethyl methacrylate
PDMS	Poly(dimethylsoloxane)
PE	Polyethylene
PEG	Poly(ethylene glycol)
PFPQ	Polyfluorene derivative
PGA	Penicillin G amidase
PHEMA	Poly(2-hydroxyethyl methacrylate)
PHMB	Polyhexamethylene biguanide
PP	Polypropylene
PS	Photosensitizer
PTPB	(4-penten-1-yl) triphenylphosphonium bromide
PU	Polyurethane
PVAms	Poly(vinyl amines)
PVP	Poly(vinyl pyrrolidone)
QAS	Quaternary ammonium salts
QPM	Tributyl(4-vinylbenzyl)phosphonium
QPS	Quaternary phosphonium salts
RAFT	Reversible addition-fragmentation chain transfer
RDRP	Reversible deactivation radical polymerization
ROS	Reactive oxygen species
SSNa	Sodium 4-styrene sulfonate
TMC	*N*,*N*,*N*-trimethyl chitosan
VBCHAM	Vinyl benzyl dimethylhexadecylammonium chloride
WHO	World Health Organization
